# Influence of kinesiophobia on pain intensity, disability, muscle endurance, and position sense in patients with chronic low back pain—a case-control study

**DOI:** 10.1186/s13063-022-06406-6

**Published:** 2022-06-06

**Authors:** Praveen Kumar Kandakurti, Watson Arulsingh, Sharad S Patil

**Affiliations:** 1 College of Health Sciences, Gulf Medical University, Ajman, United Arab Emirates; 2 Thumbay Physical Therapy & Rehabilitation Hospital, Ajman, United Arab Emirates

**Keywords:** Low back pain, Kinesiophobia, Position sense, Endurance

## Abstract

**Background:**

Patients with chronic low back pain (CLBP) frequently present with kinesiophobia. Though large body of evidence reported the impact of kinesiophobia in patients with CLBP, there are paucity of studies in associating kinesiophobia to muscle endurance and position sense in patients with CLBP. The primary aim of the study is to compare the impact of kinesiophobia on lumbar extensor endurance, position sense in patient with CLBP, and asymptomatic individuals. Secondarily, we aim to examine the association between kinesiophobia and lumbar extensor endurance, position sense, pain intensity, and functional ability in patients with CLBP. Thirdly, we aim to assess the degree of association of various factors on CLBP, lumbar endurance, and position sense.

**Material and methods:**

This case-control study will have 200 patients with CLBP and 400 controls. Kinesiophobia, lumbar endurance, and lumbar position sense will be assessed with Tampa Scale, Soren’s lumbar extensor test, and lumbar repositioning test respectively. Secondarily, the pain intensity will be assessed with visual analog scale and functional ability with Patient-specific Functional Scale in patients with CLBP. Lumbar endurance and joint position sense will be compared between subjects with and without kinesiophobia. Kinesiophobia scores will be compared with lumbar extensor endurance and proprioception joint position errors, pain intensity, and functional ability. Simple and multiple binary logistic regression will be used to determine crude and adjusted odd’s ratio for kinesiophobia, lumbar position sense and kinesiophobia, and lumbar endurance.

**Discussion:**

The finding from this study can be generalized as this study has adequate sample size and subgroup analysis by adjusting the variables to draw a valid conclusion. The finding of this study will help the working physician to include assessment of kinesiophobia as part of musculoskeletal evaluation for patient with CLBP in a prospective diagnostic intervention.

**Trial registration:**

ClinicalTrials.gov NCT05079893. Registered on 14 October 2021.

**Supplementary Information:**

The online version contains supplementary material available at 10.1186/s13063-022-06406-6.

## Background

Chronic lower back pain (CLBP) is one of leading causes of disability for adults of working age [1]. Globally, years lived with disability caused by low back pain increased by 54% between 1990 and 2015 [2]. Chronic lower back pain is defined by the location of pain between the lower rib margins and the buttock that lasts for more than 12 weeks [3, 4]. There are several causes for CLBP, and the differential diagnosis can be challenging. Specific causes for LBP compromises 15% of all back pain caused by a specific pathophysiologic mechanism, such as herniated nuclei pulposus, infection, osteoporosis, rheumatoid arthritis, fracture, or tumor [5–7]. The majority of remaining patients are labelled as having nonspecific low back pain [NSLBP]. Because NSLBP does not have pathoanatomical cause, treatment focuses on only reducing pain and further consequences [8]. Furthermore, in chronic LBP, there is also a problem in the pattern of muscular activation that influences the brain and vice versa. Despite the aspect of pain perception, there is maladaptive plasticity in chronic low back pain patients could be associated with disorders of volitional activation of trunk/pelvis muscles and alterations of their anticipatory motor patterns for postural control [9, 10]. This might lead to asymmetrical changes in the cross-sectional area of the multifidus [MF] muscles located at the lumbar spine [11].

Though there are numerous biological factors that contribute to LBP, psychological factors may play an unexpectedly large role in some patients with chronic LBP [12]. New insights are coming from different fields of research, with a lot of work being done in searching for the factors involved in persistent back pain [12]. There are many established factors such as physical, biological, cognitive, behavioral, social, and occupational associated with poor prognosis following the onset of musculoskeletal pain [13–15]. Hence, the recent recommended treatment approach is to discourage use of pain medication, steroid injections, and spinal surgery and instead promote physical and cognitive behavioral therapy [16]. Evidences support that psychological factor in the form of kinesiophobia negatively influences many treatment effects for patients with CLBP [17, 18].

Kinesiophobia is described as an excessive, unreasonable, and crippling fear of performing a physical motion due to a feeling of vulnerability to a painful injury or reinjury [19]. Clinical studies suggest that an exaggerated negative cognitive response toward actual pain known as pain catastrophizing and fear of movement/(re)injury (kinesiophobia) is important in the etiology of chronic low back pain and associated disability [20]. These findings are consistent with a cognitive-behavioral perspective that underscores the importance of maladaptive interpretations of bodily sensations [20].

The mechanism can be described as follows: persons who catastrophically misinterpret innocuous bodily sensations, including pain, are likely to become fearful of pain, which results in at least two processes. First, pain-related fear is associated with avoidance behaviors and the avoidance of movement and physical activity in particular. Avoidance also means withdrawal from rewarding activities such as work, leisure, and family. Second, pain-related fear is associated with increased bodily awareness and pain hypervigilance. Hypervigilance, depression, and disuse are known to be associated with increased pain levels and hence might exacerbate the painful experience. This model is used to develop successful treatments [20–22].

Thus, kinesiophobia is correlated to pain-related interference and triggers motor activity changes that influence activities related to pain and pain-related disability management and control [18]. The prevalence of kinesiophobia in chronic pain varies from 50 to 70% [23]. A higher degree of kinesiophobia is correlated with higher levels of perceived pain [24] and lower level re-entry into pre-injury activities for all [CMP] chronic musculoskeletal pain conditions [25]. Thus, clinicians should consider kinesiophobia as an important factor in their preliminary assessment of patients with chronic LBP [18].

Kinesiophobia may produce a wide variety of physical and psychological effects that indirectly influence back pain to be maintained or recur or cause changes in the somatosensory system [26–28]. Studies in the past reported that patients with LBP had poorer ability to sense a change in lumbar position than control subjects [29–33]. Evidence reported that higher levels of pain-related fear are significantly associated with reduced amplitudes of movement and larger muscle activity and were consistent across subgroup and moderation analyses [27, 34]. Recent systematic reviews reported a moderate to strong evidence of associations between a greater degree of kinesiophobia, disability, and poorer quality of life in CMP. However, there was lack of consensus as there was heterogeneity present between all included studies in the systematic reviews in terms of population, outcome measures, pain conditions, and statistical parameters. Hence, an efficient comparison could not be made between their included studies. Moreover, none of the included studies specifically evaluated the possible mediating effect of kinesiophobia in CMP. Furthermore, confounding variables were not always explored in all included studies [18, 35]. Recent researches exploring the influence of kinesiophobia on CLBP and its various outcome are in the infant stage.

Chronic LBP is also associated with decreased endurance of the trunk extensor muscles [TE]. In addition, studies reported TE in chronic LBP participants to be significantly weaker than asymptomatic participants. The multi-factorial dysfunctions consistently reported in literature is the deconditioning of the lumbar extensor musculature, i.e., thoracic and lumbar erector spinae, multifidus, and quadratus lumborum [36, 37].

Long-term mechanical low back pain (LBP) results in inhibition and atrophy of the deep segmental muscles such as multifidus and overactivity of the longer superficial muscles of the trunk with resultant decreased dynamic activity and increased fatigability [38, 39]. Though the muscular endurance is reported to be affected in the patient with CLBP, no studies have explored the impact of kinesiophobia on lumber muscle endurance in patient with CLBP [40, 41] against asymptomatic subjects.

Moreover, in the spine, proprioceptive information is provided by structures present in the spinal ligaments, facet joints, intervertebral discs [42], and paraspinal muscles [43]. Muscle spindle density is high in deep paraspinal rotators, which are small muscles spanning one or two segments of the spine act as kinesthetic sensors that monitor trunk position and movement. It is these muscle receptors that are more likely responsible for information in the midrange of trunk motions [44]. Deficits in proprioception in lower back pain [LBP] have generally been attributed to impaired afference from paraspinal muscle spindle or changes to its central processing. Trials with visual feedback demonstrated that participants with and without CLBP could perform the task accurately. Yet, when visual feedback was removed, participants with CLBP matched the force less accurately than control participants and undershot the target force [42–44].

To the best of our knowledge, till date there are no studies assessing the relationship between kinesiophobia, lumbar endurance, and lumbar position sense in subjects with CLBP. Therefore, the current study aims to compare the impact of kinesiophobia on lumbar extensor endurance, and position sense in patient with CLBP and asymptomatic individuals. Secondarily, we aim to examine the association between kinesiophobia and lumbar extensor endurance, position sense, pain intensity, and functional ability in patients with CLBP. Thirdly, we aim to assess the degree of association with various factors on CLBP, lumbar endurance, and position sense. Therefore, we hypothesized that kinesiophobia would impact the lumbar position sense and lumbar endurance as well.

## Methods

This is a case-control study where the patients with CLBP conditions and asymptomatic controls will be recruited from Thumbay Physical Therapy & Rehabilitation Hospital, Gulf Medical University, Ajman, United Arab Emirates. After obtaining ethical clearance, informed consent will be taken from all the recruited participants. The recruiting of participants for this study will be done from May 2022 to November 2022.

### Sample size

As for the calculation of the sample size, the odds ratio taken is 2 with the prevalence of 70% kinesiophobia [17] among CLBP patients and assumed the prevalence of 10% kinesiophobia among the controls, with the minimum required sample size of 600 [200 cases + 400 controls]. The case-control ratio taken is 1:2. The controls will be invited participants/patients who have no complaints of lower back pain at the Thumbay Physical Therapy & Rehabilitation Hospital, Ajman. All possible confounding factors will be controlled through subgroup analysis in later part during data analysis. The patients and controls will be identified from the outpatient clinic prospectively.

### Inclusion criteria for CLBP subjects

- Adults aged between 18 and 59 years [45].
- Patient suffering from low back pain for at least 3 months and referred by an orthopedic doctor or general physician.
- Participants with enough physical autonomy to take part in physical activities such as performing lumbar endurance and lumbar positioning test required of the study.

### Exclusion criteria CLBP subjects

- Low back pain patients with neurological deficit, any neurological disorder, post spinal fractures, history of spinal tumors, cauda equina syndrome, tuberculosis spine, any congenital spine anomalies, vestibular issue, joint instability in lower limb, cardiorespiratory problem, and hip arthritis.
- Patient under antidepressive medication and antihypertensive medication
- Patient unwilling to participate

### Inclusion criteria for asymptomatic subjects

- Adults aged between 18 and 59 years.
- Either gender.

### Exclusion criteria for asymptomatic subjects

- History of (H/o) previous lower back injury.
- Participants with congenital spine anomalies, vestibular issue, joint instability in lower limb, cardiorespiratory problem, and hip arthritis.
- H/o inflammatory, infectious disease, and malignancy in the spine
- Patient under antidepressive medication and antihypertensive medication

### Procedures

The initial screening of participants will be performed by a physiotherapist (examiner 1) during the first physical therapy appointment. Then, the participants will be instructed to fill a Tampa Scale for kinesiophobia which is a questionnaire consisting of socio-demographic information (age, gender, weight, height), pain characteristics (pain area and associated symptoms); fear-avoidance behavior and physical examination carried out by the examiner. The evaluation of pain intensity and function will be performed through self-reported questionnaires (visual analog scale—VAS and Patient-specific Functional Scale). The physiotherapist (examiner 2) who carries out the lumbar endurance and position sense will be blinded for the cases and control group.

### Outcome measures

**Fig. 1:**
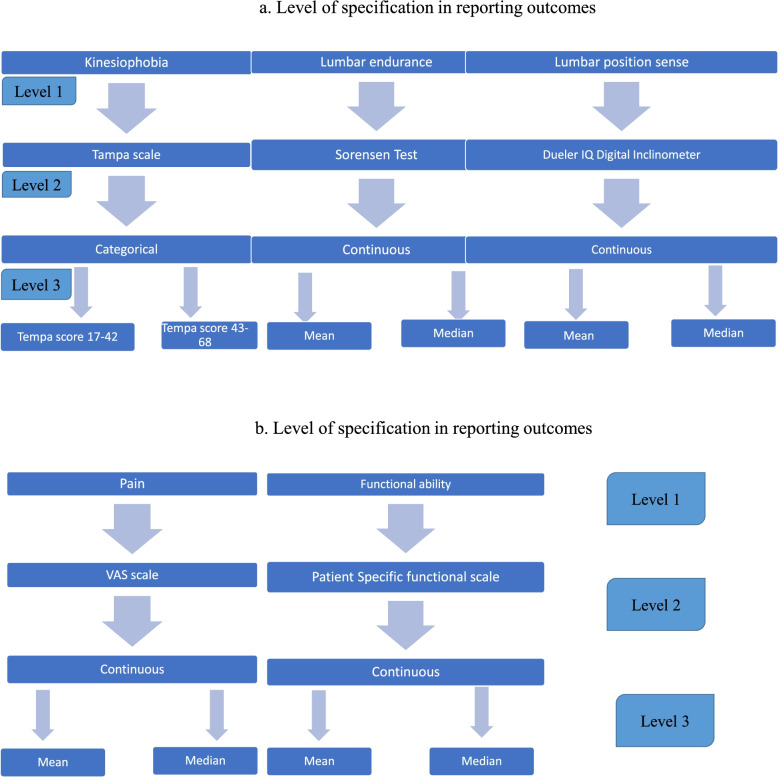
**a**, **b** Level of specification in reporting outcome

Three levels of specifications in reporting five outcome measures to be used in this study are provided in Fig. 1a, b.

#### Kinesiophobia

Fear of movement/injury or reinjury will be assessed using the Tampa Scale for Kinesiophobia (TSK), a scale with 17 self-reporting items with scores ranging from 17 (absence of fear) to 68 (highest fear) [46]. Tampa Scale for Kinesiophobia has been reported to have good reliability in patients with CLBP [47].

#### Lumbar extensor endurance

All these experiments will be conducted in a laboratory setting. The subjects will be tested during a 1-h session and will be asked to undergo a body weight-dependent isometric back extension (Sorensen) test on a horizontal table [48]. Sorensen test will be performed in the prone position, with the iliac crests aligned with the table edge and the lower limbs fixed by straps at the ankles and below the knees. During the test, the participants will be instructed to keep their body (head, arms, and trunk) unsupported, horizontal to the ground, as long as they could, with their arms crossed at the chest [48]. To maintain the horizontal position throughout the test, the investigator will give them verbal feedback, and the test will be ended when they could not hold the test position. Verbalized encouragement will be provided throughout the test. The participants will be instructed to maintain the lumbar lordosis position as stable as possible. The endurance will be recorded by investigator with the help of a stopwatch in seconds. A chair with cushioned seat (or with a pillow over the seat) will be placed in front of the subject so that he can support himself if fatigued during the test. The stopwatch will be stopped as soon as the subject gets fatigued or can no longer sustain the position [48].

#### Lumbar repositioning tests

Subjects will be instructed not to perform any strenuous physical activity for 24 h prior to testing and to not drink or eat 2 h prior to testing (to minimize cutaneous input from a distended abdomen). For testing, subjects will be blindfolded to eliminate visual input and the room will be kept quiet to limit auditory input. Subjects will be asked to stand in a neutral position, with their knees straight and weight equally on both feet. The primary sensor digital inclinometer will be placed over the lateral chest (T12 level) and secondary sensor over the hemi-pelvis (S1 level) in the sagittal plane to measure lumbar reposition errors in flexion. A primary sensor (T12) and secondary sensor (sacral midpoint) in the frontal plane will be used to record lateral bending angle error. Velcro straps will be used to secure the digital inclinometer for testing. Dualer IQ digital inclinometers (DIs; J-Tech Medical, Midvale, UT, USA) will be used to measure lumbar joint position error. Digital inclinometers are reliable, fast, and high in measurement precision and allow clinicians to evaluate ROM and proprioception using dynamic inclinometry like that used in other goniometric protocols [49]. The digital inclinometers have shown test-retest reliability for measuring spinal ROM, and evaluation protocols are well established and endorsed by the American Medical Association (AMA) [49].

For neutral lumbar positioning (NLP) testing, subjects will be asked to maintain the lumbar spine in a neutral position with their eyes closed. The inclinometer will be calibrated to a starting position (0 degrees) by the examiner [49]. The subjects will be asked to memorize this neutral position for few seconds, perform active full flexion, and then relocate to neutral position. Subjects will be instructed to perform the test as accurately as possible and indicate verbally when they thought they had returned to the starting position. Relocation accuracy will be measured in degrees. The NLP test will be performed in one direction only (lumbar flexion) [49].

For target lumbar positioning (TLP), the examiner guided in a slow steady pace that subject’s lumbar spine reached to a predetermined target, 50% of the maximum ROM. This range will be chosen so that all subjects could achieve it. The spine will be maintained in the target position for 5 s, subjects will be asked to remember the position, and the lumbar spine will be guided to a neutral position [49]. Subjects will then be asked to actively reposition by bending the spine to the target position. When the subjects reach the reference position, relocation accuracy will be measured in degrees [49]. Subjects will perform a total of three trials for each movement direction (flexion, lateral bending). The average of three trials will be used for analysis. The order in which movement directions will be tested will be randomized using a simple lottery method. Only the absolute error will be taken as a measurement; absolute error is the difference between the actual angles relative to the target angle and thus has no directional bias when compared to constant error or relative error.

#### Pain

Pain will be measured using a visual analog scale (VAS). The visual analog scale consists of a 10-cm line, with the left extremity representing (absence of pain) and the right extremity indicating (great pain) [50]. As per the recent evidence, either VAS or NRS scores are the validated predictors of the disability in CLBP conditions [51]. Participants were asked to indicate in the scale their current level of pain, higher values being related to more intense pain [52].

#### Functional ability

Functional ability will be measured using Patient-specific Functional Scale [PSFS] where patients are asked to identify up to five important activities they are unable to perform or are having difficulty with as a result of their problem, e.g., putting socks on, shopping. Patients are asked to rate (on an 11-point scale) the current level of difficulty associated with each activity, where “0” represents “unable to perform” and “10” represents “able to perform at prior level.” Patients select a value that best describes their current level of ability on each activity assessed [53]. The PSFS can be used with confidence for measuring change over time in individual patients within a limited range of musculoskeletal conditions, including knee, low back, or neck dysfunction [54].

The PECO model is provided for the reader below for comprehensive understanding of this study.

### PECO model for this study

- P—Patient with chronic lower back pain
- E—Primarily kinesiophobia and other confounding factors
- C—Asymptomatic subjects
- O—Lumbar position sense, lumbar muscle endurance, pain, and functional ability

Lifestyle factors, such as excess weight, physical inactivity, poor diet, and smoking, are linked to low back pain chronicity and disability. Smoking was categorized as non-smoker, often inhale secondhand smoke, quit smoking in less than 1 year, and smoker. Alcohol consumption was divided as not drinking, ≤ 1 glass/week, 2–3 glasses/week, and ≥ 4 glasses/week. Physical activity including any form of physical exercise was categorized as none, ≤ 2 h/week, 3–6 h/week, and ≥ 7 h/week. Dietary habits were categorized into 3 groups: (1) intake of no or low vegetables with high meat, (2) intake of moderate vegetables with moderate meat, and (3) intake of high vegetables with no or low meat [55, 56].

### Statistical analysis

The statistical analysis will be computed using SPSS Statistics software, version 27 (IBM, Chicago, IL, USA). The data analyses will be conducted by a statistician. Continuous variables will be summarized using standard measures of central tendency and dispersion, either as mean and standard error, or median and interquartile range. Dichotomous or categorical variables will be summarized by frequencies or denominators and percentage.

Kinesiophobia, lumbar endurance, and joint position sense will be compared between patients and controls using appropriate statistical tools. Kinesiophobia scores will be correlated with lumbar extensor endurance and proprioception joint position errors, pain intensity, and functional ability. Chi-square test will be used to find the association between kinesiophobia and lumbar endurance and joint position sense. Spearman rank correlation will be used to find the correlation between kinesiophobia, pain, and functional ability. Simple and multiple binary logistic regression will be used to determine crude and adjusted odds ratio for kinesiophobia, lumbar position sense and kinesiophobia, and lumbar endurance.

### Clinical significance/ impact of the study

- The current study can be helpful to identify whether kinesiophobia has any impact on the characterizes of patient with CLBP.
- The current study can be helpful to guide physician whether kinesiophobia be part of evaluation of patient with CLBP.

## Discussion

This study is to be conducted in Thumbay Physical Therapy & Rehabilitation Hospital which is equipped with all advanced equipment necessary for physiotherapy assessment associated with this study. Sample recruitment is expected to start from May 25, 2022, at Thumbay Physical Therapy & Rehabilitation Hospital, and Thumbay University Hospital and Gulf medical University, Ajman, United Arab Emirates.

Screening of individuals with and without chronic low back pain will be carried out by 4 experienced physiotherapists [2 males and 2 females] who are specialized in musculoskeletal and sports physiotherapy. In order to make the data collection uniform, all the therapists will be provided with the information about the study protocol including assessment methods using various assessment tools related to this study.

As per the medical record obtained on the prevalence of lower back pain conditions in Thumbay Physical Therapy & Rehabilitation Hospital and Thumbay University Hospital, we assume that the estimated sample size can be achieved within the study period given in the trial. Furthermore, the investigators of the study will contact the various departments of Thumbay University Hospital to refer the patients with and without low back pain to meet the sample required.

At least 80% of samples from the estimated sample size is anticipated for statistical analysis and reporting the study findings. Since the study covers a wide range of populations from various countries reporting to these medical centers, addressing confounding variables makes the study findings novel and generalizable.

## Trail status

ClinicalTrials.gov Identifier: NCT05079893 Registered on 14/10/2021

## Supplementary Information

**Additional file 1.** Proforma[Data entry].

## Data Availability

All authors will have access to the final trial dataset.

## References

[1] Murray CJL, Atkinson C, Bhalla K (2013). The state of US health, 1990-2010: burden of diseases, injuries, and risk factors. JAMA.

[2] Buchbinder R, van Tulder M, Öberg B (2018). Low back pain: a call for action. Lancet.

[3] Deyo RA, Weinstein JN (2001). Low back pain. N Engl J Med.

[4] Vadalà G, Russo F, Musumeci M, D’Este M, Cattani C, Catanzaro G, Tirindelli MC, Lazzari L, Alini M, Giordano R (2017). Clinically relevant hydrogel-based on hyaluronic acid and platelet rich plasma as a carrier for mesenchymal stem cells: rheological and biological characterization. J Orthop Res.

[5] Amirdelfan K, McRoberts P, Deer TR (2014). The differential diagnosis of low back pain: a primer on the evolving paradigm. Neuromodulation Technol Neural Interface.

[6] Koes BW, van Tulder MW, Thomas S (2006). Diagnosis and treatment of low back pain. Br Med J.

[7] Maher C, Underwood M, Buchbinder R (2017). Non-specific low back pain. Lancet.

[8] Negrini S, Zaina F (2013). The chimera of low back pain etiology: a clinical rehabilitation perspective. Am J Phys Med Rehabil.

[9] Lamoth CJC, Meijer OG, Daffertshofer A (2006). Effects of chronic low back pain on trunk coordination and back muscle activity during walking: changes in motor control. Eur Spine J.

[10] Massé-Alarie H, Schneider C (2011). Cerebral reorganizationin chronic low back pain and neurostimulation to improve motor control [in French]. Neurophysiol Clin.

[11] Li X, Liu H, Ge L, Yan Y, Lo WLA, Li L, Wang C (2021). Cortical representations of transversus abdominis and multifidus muscles were discrete in patients with chronic low back pain: evidence elicited by TMS. Neural Plast.

[12] Hoy D, Brooks P, Blyth F, Buchbinder R (2010). The epidemiology of low back pain. Best Pract Res Clin Rheumatol.

[13] Cimmino MA, Ferrone C, Cutolo M (2011). Epidemiology of chronic musculoskeletal pain. Best Pract Res Clin Rheumatol.

[14] Artus M, Campbell P, Mallen CD (2017). Generic prognostic factors for musculoskeletal pain in primary care: a systematic review. BMJ Open.

[15] Gatchel RJ, Peng YB, Peters ML (2007). The biopsychosocial approach to chronic pain: Scientific advances and future directions. Psychol Bull.

[16] Traeger AC, Buchbinder R, Elshaug AG, Croft PR, Maher CG (2019). Care for low back pain: can health systems deliver?. Bull World Health Organ.

[17] Uluğ N, Yakut Y, Alemdaroğlu İ, Yılmaz Ö (2016). Comparison of pain, kinesiophobia and quality of life in patients with low back and neck pain. J Phys Ther Sci.

[18] Luque-Suarez A, Martinez-Calderon J, Falla D (2019). Role of kinesiophobia on pain, disability and quality of life in people suffering from chronic musculoskeletal pain: a systematic review. Br J Sports Med.

[19] Kori SH, Miller RP, Todd DD. Kinisophobia: a new view of chronic pain behavior. Pain Management. 1990;3:35–43.

[20] Fritz JM, George SZ, Delitto A (2001). The role of fear-avoidance beliefs in acute low back pain: relationships with current and future disability and work status. Pain.

[21] van Tulder MW, Ostelo RW, Vlaeyen JWS (2001). Behavioral treatment for chronic low back pain. A systematic review within the framework of the Cochrane Back Review Group. Spine.

[22] Linton SJ, Andersson T (2000). Can chronic disability be prevented? A randomized trial of a cognitive-behavior intervention for patients with spinal pain. Spine.

[23] Larsson C, Hansson EE, Sundquist K, Jakobsson U (2014). Psychometric properties of the Tampa Scale of Kinesiophobia (TSK-11) among older people with chronic pain. Physiother Theory Pract.

[24] Palomo-López P, Becerro-de-Bengoa-Vallejo R, Losa-Iglesias ME, López-López D, Rodríguez-Sanz D, Romero-Morales C (2020). Kinesiophobia and pain intensity are increased by a greater hallux valgus deformity degree-kinesiophobia and pain intensity in hallux valgus. Int J Environ Res Public Health.

[25] Chmielewski TL, Jones D, Day T, Tillman SM, Lentz TA, George SZ (2008). The association of pain and fear of movement/reinjury with function during anterior cruciate ligament reconstruction rehabilitation. J Orthop Sports Phys Ther.

[26] Aydoğdu O, Zübeyir S (2020). The association between kinesiophobia and proprioception, postural stability, activity level, knee function, and quality of life following anterior cruciate ligament reconstruction. J Exerc Ther Rehabil.

[27] Ishak NA, Zahari Z, Justine M (2017). Kinesiophobia, pain, muscle functions, and functional performances among older persons with low back pain. Pain Res Treat.

[28] Freigang V, Müller K, Ernstberger A, Kaltenstadler M, Bode L, Pfeifer C (2020). Reduced recovery capacity after major trauma in the elderly: results of a prospective multicenter registry-based cohort study. J Clin Med.

[29] Taimela S, Kankaanpaa M, Luoto S (1999). The effect of lumbar fatigue on the ability to sense a change in lumbar position. A controlled study. Spine.

[30] Bailey KM, Carleton RN, Vlaeyen JW, Asmundson GJ (2010). Treatments addressing pain-related fear and anxiety in patients with chronic musculoskeletal pain: a preliminary review. Cogn Behav Ther.

[31] Picavet HSJ, Vlaeyen JW, Schouten JS (2002). Pain catastrophizing and kinesiophobia: predictors of chronic low back pain. Am J Epidemiol.

[32] Wand BM, James M, Abbaszadeh S (2014). Assessing self-perception in patients with chronic low back pain: development of a back-specific body-perception questionnaire. J Back Musculoskelet Rehabil.

[33] Neblett R, Hartzell MM, Williams M (2017). Use of the Central Sensitization Inventory (CSI) as a treatment outcome measure for patients with chronic spinal pain disorder in a functional restoration program. Spine J.

[34] Christe G, Crombez G, Edd S, Opsommer E, Jolles BM, Favre J (2021). Relationship between psychological factors and spinal motor behaviour in low back pain: a systematic review and meta-analysis. Pain.

[35] Luque-Suarez A, Falla D, Morales-Asencio JM, Martinez-Calderon J (2020). Is kinesiophobia and pain catastrophising at baseline associated with chronic pain and disability in whiplash-associated disorders? A systematic review. Br J Sports Med.

[36] Roy SH, De Luca CJ, Emley M, Buijs RJ (1995). Spectral electromyographic assessment of back muscles in patients with low back pain undergoing rehabilitation. Spine.

[37] Nelson BW, Miller M, Hogan M, Wegner JA, Kelly C (1995). The clinical effects of intensive, specific exercise on chronic low back pain: a controlled study of 895 consecutive patients with 1-year follow up. Orthopedics.

[38] Mbada CE, Ayanniyi O, Ogunlade SO, Orimolade EA, Oladiran AB, Ogundele AO (2013). Rehabilitation of back extensor muscles’ inhibition in patients with long-term mechanical low-back pain. Int Sch Res Notices.

[39] Conway R, Behennah J, Fisher J, Osborne N, Steele J (2016). Associations between trunk extension endurance and isolated lumbar extension strength in both asymptomatic participants and those with chronic low back pain. Healthcare (Basel).

[40] Suuden E, Ereline J, Gapeyeva H, Paasuke M (2008). Low back muscle fatigue during Sorensen endurance test in patients with chronic low back pain: relationship between electromyographic spectral compression and anthropometric characteristics. Electromyogr Clin Neurophysiol.

[41] Boyce RO, Boone E, Stallings J, Wilde C (2008). A multidisciplinary approach to a time efficient low back exercise intervention in a small manufacturing plant: a case study. J Exerc Physiol.

[42] Holm S, Indahl A, Solomonow M (2002). Sensorimotor control of the spine. J Electromyogr Kinesiol.

[43] Brumagne S, Cordo P, Lysens R, Verschueren S, Swinnen S (2000). The role of paraspinal muscle spindles in lumbosacral position sense in individuals with and without low back pain. Spine.

[44] Solomonow M (2006). Sensory - motor control of ligaments and associated neuromuscular disorders. J Electromyogr Kinesiol.

[45] Meucci RD, Fassa AG, Faria NM (2015). Prevalence of chronic low back pain: systematic review. Rev Saude Publica.

[46] Rosenbloom BN, Pagé MG, Isaac L (2020). Fear of movement in children and adolescents undergoing major surgery: a psychometric evaluation of the Tampa Scale for Kinesiophobia. Eur J Pain.

[47] Schouppe S, Clauwaert A, Van Oosterwijck J (2020). Does experimentally induced pain-related fear influence central and peripheral movement preparation in healthy people and patients with low back pain?. Pain.

[48] Behennah J, Conway R, Fisher J (2018). The relationship between balance performance, lumbar extension strength, trunk extension endurance, and pain in participants with chronic low back pain, and those without. Clin Biomech.

[49] Alahmari KA, Reddy RS, Samuel PS, et al. Intra-rater and inter-rater reliability of neutral and target lumbar positioning tests in subjects with and without non-specific lower back pain. J Back Musculoskel Rehabil. 2020:1–11. 10.3233/BMR-200010 33285625

[50] Couper M, Tourangeau R, Conrad F (2006). Evaluating the effectiveness of visual analog scales: a web experiment. Soc Sci Comput Rev.

[51] Shafshak TS, Elnemr R (2021). The visual analogue scale versus numerical rating scale in measuring pain severity and predicting disability in low back pain. J Clin Rheumatol.

[52] Mikhail H, Reda N, Shahaly M, Nour-Eldein H. Predictors of fear-avoidance belief, pain, and disability index in patients with chronic low back pain attending rheumatology outpatient clinics. J Public Health. 2022;30. 10.1007/s10389-020-01296-x.

[53] Stratford P, Gill C, Westaway M, Binkley J (1995). Assessing disability and change on individual patients: a report of a patient specific measure. Physiother Can.

[54] Horn KK, Jennings S, Richardson G, Vliet DV, Hefford C, Abbott JH (2012). The patient-specific functional scale: psychometrics, clinimetrics, and application as a clinical outcome measure. J Orthop Sports Phys Ther.

[55] Robson EK, Kamper SJ, Davidson S (2019). Healthy Lifestyle Program (HeLP) for low back pain: protocol for a randomised controlled trial. BMJ Open.

[56] Muga MA, Owili PO, Hsu CY (2019). Association of lifestyle factors with blood lipids and inflammation in adults aged 40 years and above: a population-based cross-sectional study in Taiwan. BMC Public Health.

