# Supplementary material for: Influence of kinesiophobia on pain intensity, disability, muscle endurance, and position sense in patients with chronic low back pain—a case-control study
Source: Trials. 2022 Jun 6;23:469. doi: 10.1186/s13063-022-06406-6 (PMC9169280; doi:10.1186/s13063-022-06406-6)
Supplement: Supplementary file 1 — Additional file 1. Proforma[Data entry]. [file 13063_2022_6406_MOESM1_ESM.docx]

**Additional file 1** Proforma[Data entry]

| Name of the participant |  |
| --- | --- |
| Type -Patient/Control |  |
| Female |  |
| Age (years) |  |
| BMI (kg/m^2^) |  |
| Currently employed |  |
| **Lifestyle** | |
| Smoking-  non-smoker/often inhale second hand smoke/ quit smoking in less than 1 year and smoker |  |
| Alcohol consumption- ≤ 1 glass/week, 2–3 glasses/week and ≥ 4 glasses/week |  |
| Physical activity- none, ≤ 2 h/week, 3–6 h/week and ≥ 7 h/week |  |
| Dietary habits- intake of no or low vegetables with high meat, (2) intake of moderate vegetables with moderate meat and (3) intake of high vegetables with no or low meat |  |
| Duration of CLBP |  |
| Presence of leg pain Yes/No |  |
| Household income/week (year) (AED) |  |
| Patient takes simple analgesics Yes/No |  |
| Patient takes strong opioid analgesics Yes/No |  |
| Patient takes combination opioid analgesics Yes/No |  |
| Other |  |
| **Occupation** |  |
| Manager/ Technician/ Trade Worker/ Clerical/Driver/ Machinery Operator /Professional/other |  |
| **Health insurance** | |
| None/Yes |  |
| **Episode characteristics** |  |
| Dermatomal pain [yes/no] |  |
| Motor deficit [yes/no] |  |
| Sensory deficit [yes/no] |  |
| **Clinician diagnosis** |  |
| Spinal level affected -L3,L4 |  |
| Spinal level affected -L4,L5 |  |
| Kinesiophobia score |  |
| Lumbar endurance score |  |
| Lumbar position sense measure |  |
| Visual analogue scale (pain) score |  |
| Patient specific functional scale score |  |

Evaluator Date:
